# Adherence to follow‐up after the exit cervical cancer screening test at age 60–64: A nationwide register‐based study

**DOI:** 10.1002/cam4.4420

**Published:** 2021-11-12

**Authors:** Susanne F. Jørgensen, Berit Andersen, Lone Kjeld Petersen, Matejka Rebolj, Sisse H. Njor

**Affiliations:** ^1^ University Research Clinic for Cancer Screening Department of Public Health Programmes Randers Regional Hospital Randers Denmark; ^2^ Department of Clinical Medicine Aarhus University Aarhus Denmark; ^3^ Department of Gynaecology and Obstetrics Odense University Hospital Odense Denmark; ^4^ Open Patient Data Explorative Network (OPEN) University of Southern Denmark Odense Denmark; ^5^ Cancer Prevention Group School of Cancer & Pharmaceutical Sciences Faculty of Life Sciences & Medicine King's College London London UK

**Keywords:** elderly women, follow‐up, guideline adherence, HPV screening

## Abstract

**Background:**

In Denmark, human papillomavirus (HPV) testing has replaced cytology in primary cervical cancer screening for women aged 60–64; at this age, women are invited for the last (exit) screening test within the national organized program.

**Aim:**

We investigated the adherence of these women to the recommended follow‐up after a non‐negative (positive or inadequate) HPV test and the overall resource use during that follow‐up.

**Materials & Methods:**

We included all 2926 women aged 60–64 years with nonnegative HPV screening tests between March 2012 and December 2016. All relevant follow‐up tests and procedures were retrieved until the end of 2020 from the highly complete Danish administrative health registers, and the data were linked at the individual level. We determined the extent to which the adherence patterns followed the national recommendations for follow‐up and estimated the total numbers of tests and diagnostic procedures utilized during the entire process.

**Results:**

In total, only 26% of women had follow‐up in accordance with the recommendations; 4% had no follow‐up, 46% had insufficient follow‐up, and 24% had more follow‐up than recommended. We estimated that 17% of women remained in follow‐up for longer than 4 years. The average numbers of diagnostic tests and procedures used after positive HPV screening were higher than expected, even among women who had insufficient follow‐up, that is, those who received less invasive procedures than recommended, or experienced delays in receiving those procedures.

**Conclusion:**

To conclude, we found that the patterns of follow‐up of women with nonnegative primary HPV screening tests at 60–64 often diverged from the recommendations. Addressing these inconsistencies in follow‐up by providing evidence for optimal clinical management should help improve the quality of screening programs and secure an equal and reliable follow‐up care service for all women.

## Graphical abstract

We mapped the entire follow‐up pathways after positive HPV exit screening among women aged 60–64 years. Only one fourth of the women complied to the national recommendations for follow‐up and we found extensively long and apparently individualized pathways. There is an urgent need for evidence‐based guidelines with clear recommendations for when to end screening and the associated follow‐up in this age group.

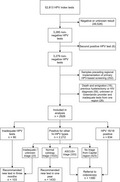

## 1 INTRODUCTION

Adequate follow‐up is crucial in preventing the development of screen‐detected abnormalities into cervical cancer.^1^ Screening guidelines usually recommend a series of additional surveillance and diagnostic tests to determine whether treatment is necessary. Despite the central role that such follow‐up plays in the success of cervical cancer screening, little is known on how well the guidelines are adhered to in terms of the timing of the tests and the overall resource use.^2^, ^3^, ^4^, ^5^, ^6^, ^7^, ^8^, ^9^

Denmark has had a long‐standing cervical cancer screening program using evidence‐based guidelines. This program is continuously monitored for its performance.^10^ It has been achieving a relatively high coverage rate (>70%), and has helped to decrease the incidence and the mortality from the disease.^2^, ^11^ Within this context, we have recently shown that 4% of Danish women with cytological abnormalities at age 23–59 received no follow‐up; for 35%, the follow‐up was less comprehensive that what the guidelines recommended; while a further 18% received more, although mostly without a benefit in the form of detecting high‐grade cervical intraepithelial neoplasia (CIN2+).^12^ This variation in how abnormalities were followed up was associated with overuse of certain health care services such as colposcopy.

Several European countries are now switching to human papillomavirus (HPV) testing as the primary screening method.^13^ In Denmark, HPV testing has been used for primary screening since 2012, but only for women aged 60–64 in their last screening round, having a so‐called “exit test.”^14^ If that test is negative, women are discharged from the program regardless of their screening history.^14^ Those with a positive test are offered a series of additional tests and treatments, until their abnormality is considered resolved. However, the definition of a resolved abnormality remains open‐ended for women with persistently positive HPV tests who have no treatable histological abnormalities. The problem of open‐ended HPV screening guidelines in this age group is shared between the different programs,^15^ and it is unknown what this means for the care that the affected women are receiving in practice.

We evaluated the adherence to the recommended follow‐up in Danish women screened with an HPV exit test during the first 4 years of the guideline implementation. We investigated how that adherence was related to the type of the screening provider and the woman's history of screening abnormalities. Finally, we also estimated the volume of diagnostic tests and procedures spent during the follow‐up.

## 2 MATERIALS AND METHODS

### 2.1 Setting

**FIGURE 1:**
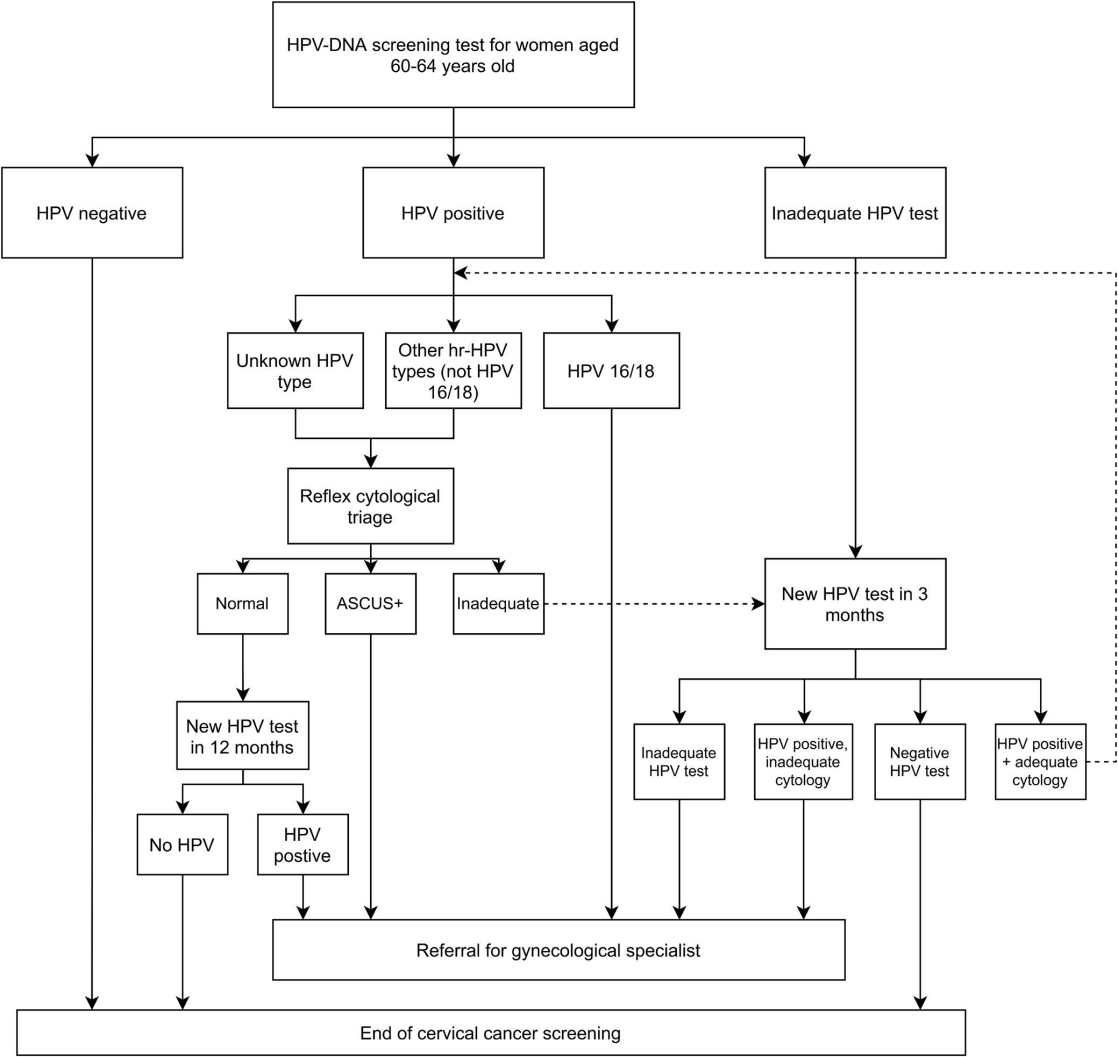
Danish national recommendations for follow‐up after primary HPV screening among women aged 60–64 years. Reference: Danish Health Authority. National recommendations of cervical cancer screening 2012 [in Danish]

**FIGURE 2:**
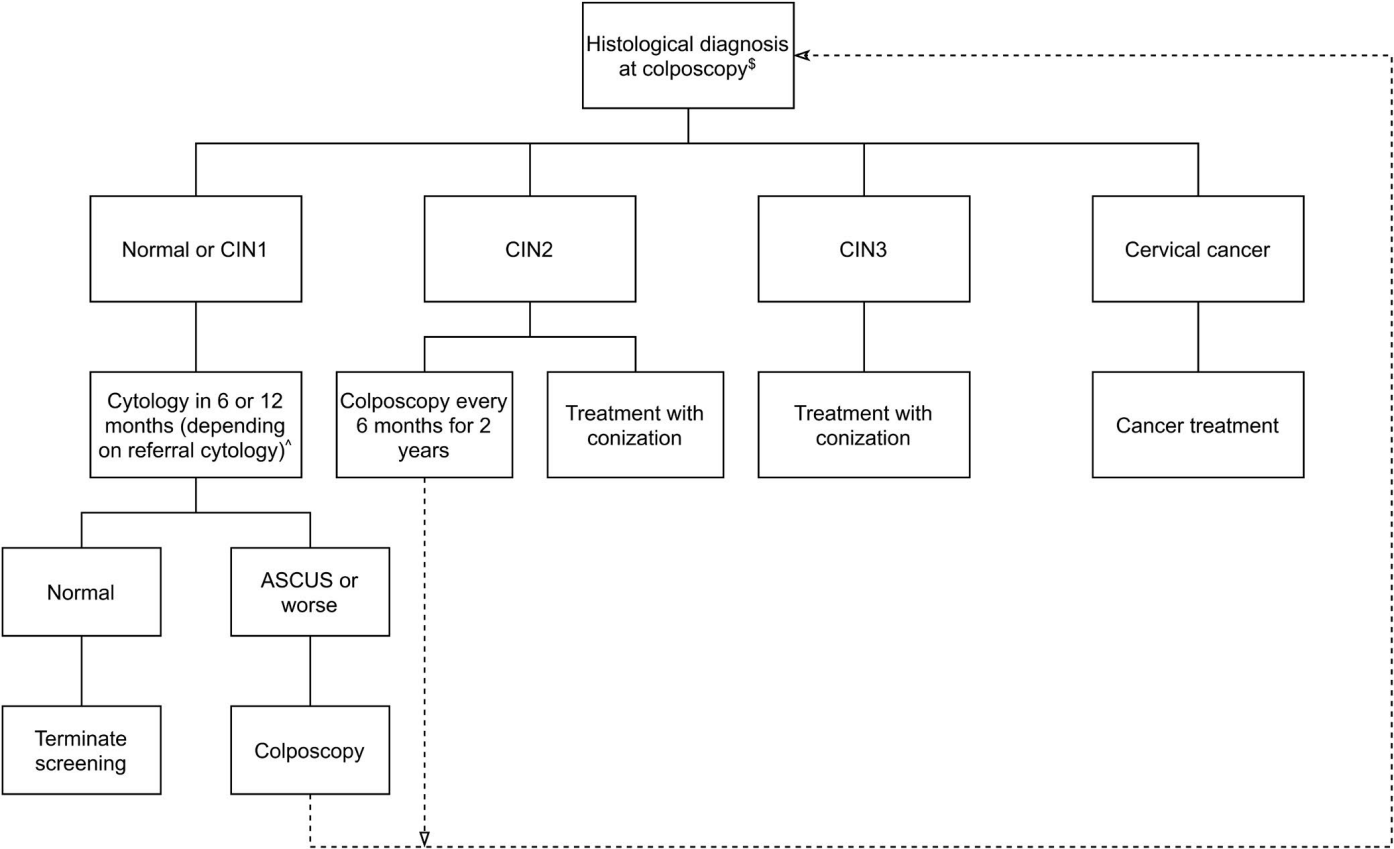
Recommendations for follow‐up after referral to colposcopy. Reference: Danish Society of Obstetrics and Gynaecology. Diagnostics, treatment and surveillance of cervical dysplasia 2012 [in Danish]

The national recommendations for screening with an HPV exit test including the follow‐up when the test is positive or inadequate were published by the Danish National Health Authority in 2012.^14^ National guidelines for follow‐up after colposcopy were published in 2012 by the Danish Society of Obstetrics and Gynaecology.^16^ See full description of the guidelines in Figures 1 and 2. Women positive for HPV genotypes 16 or 18 should be referred to colposcopy. HPV‐positive women infected with any of the other 12 high‐risk genotypes are referred to colposcopy if triage shows cytological abnormalities (defined as at least atypical squamous cells of undetermined significance, ASCUS, in squamous or glandular cells), and to re‐testing in 1 year if cytology is normal, while inadequate HPV tests are recommended to have a re‐test in 3 months. Cytology is read with knowledge of an HPV infection. Only DNA tests have been used for HPV‐based primary cervical cancer screening.

While the recommendations for screening and follow‐up are decided nationally, the program is organized by administrative region. This usually leads to some differences in when and how the guidelines are implemented. The last region to implement the new guidelines did so about 2 years after the first region (Appendix A, Figure A1), while one region (Capital) referred all HPV‐positive women to colposcopy, regardless of the infecting genotype.^17^

### 2.2 Study population

We included women aged 60–64 with a nonnegative (inadequate or a positive) HPV screening test, having their first screening test between the date that their region first implemented HPV testing (range: 1 March 2012–1 September 2014) and 31 December 2016. As the reason for taking the sample is not reliably registered, we included only women who had (1) no abnormal test, no recommendation for follow‐up, and did not opt out of screening within the preceding 2 years, (2) no biopsy registered on the same day, and (3) no previous diagnosis of cervical cancer. We excluded women who died or emigrated during follow‐up, were from Greenland, had their tests taken by an unknown provider, or had a hysterectomy or a diagnosis of HIV before the screening test. From the Capital region, we excluded women with inadequate HPV tests, as there were fewer than five (Danish legislation does not allow reporting counts smaller than five, to reduce the risk of re‐identification).

We included all follow‐up tests and diagnostic procedures reported to the registers described below, as well as their timing, until 31 December 2020. To avoid including follow‐up tests performed due to symptoms, the tests had to be performed in at most 24‐month intervals; any tests taken after a longer interval were considered to be taken for reasons other than the recommended follow‐up of the nonnegative screen. We also censored the follow‐up at treatment (either with conization, usually with large‐loop excision of the transformation zone, or hysterectomy).

### 2.3 Data sources

Information on HPV tests, cytology, biopsies, and conizations, including diagnoses, dates, and provider codes was retrieved from the National Pathology Register.^18^ In this register, the proxy for colposcopy was a sample registered with a specialist/hospital code, since colposcopies are not performed by general practitioners (GPs). Information on cervical cancer diagnoses was retrieved from the Danish Cancer Registry,^19^ while information on HIV diagnoses and hysterectomies was retrieved from the Danish National Patient Register.^20^ The population eligible for screening, together with information on deaths and emigration, was identified in the Civil Registration System. Here, all residents in Denmark are registered with personal identification numbers, which were used for linkage.^21^ All these registers are considered to be of high quality and be highly complete.^22^

### 2.4 Definition of variables

We categorized women depending on the follow‐up that they were recommended for. This resulted in three groups: (1) inadequate screening tests and samples positive for other HPV genotypes either without cytological triage or with inadequate triage cytology, recommended for a new test in 3 months; (2) screening tests positive for HPV16/18 or for other high‐risk HPV genotypes combined with abnormal cytology in four out of five regions and all HPV‐positive women in the Capital region, recommended for colposcopy due to local guidelines^17^; and (3) screening tests positive for other high‐risk HPV genotypes with normal cytology, recommended for a new HPV test in 1 year.

The follow‐up pathway of each woman was evaluated in its entirety until 31 December 2020, no new test or other diagnostic procedure in 24 months, or treatment, whichever came first. Each event was categorized in terms of the timeliness and the appropriateness according to the national recommendations (Figures 1 and 2). All women had at least 4 years of follow‐up in the available data; this should suffice for at least the first three recommended follow‐up visits. In our main analysis, therefore, we evaluated the adherence to the first three follow‐up visits. For each of the three groups of women separately, the observed follow‐up pathways were categorized into: (a) no follow‐up for at least 2 years, (b) less comprehensive follow‐up than recommended, defined by either tests that were taken too late, tests that did not include HPV testing, or follow‐up by a GP when a referral to a gynecologist was recommended, (c) follow‐up in which the type of tests and their timing were in accordance with the recommendations, and (d) more intensive follow‐up than recommended, defined by more follow‐up tests, colposcopies, or treatments than recommended. Full description of how the categorization was performed, is presented in Appendix A, Figure A2. The assessment of timeliness was made by considering reasonable delays. These depended on the length of the recommended timing of the follow‐up events (Appendix A, Table A1).

### 2.5 Persistent HPV infection and negative cytology

Independent of the categorization of adherence, we estimated the proportion of women who continued being HPV‐positive with any high‐risk genotype throughout three consecutive follow‐up tests but had no cervical abnormalities on cytology or biopsies.

### 2.6 Statistical analyses

Adherence to the recommended follow‐up was described with proportions and exact binomial 95% confidence intervals (CIs). Follow‐up time was reported as medians with interquartile ranges (IQRs). Binomial regression analyses were used to calculate relative risks (RR's) for each type of a deviation from the recommended follow‐up, compared to the probability of having received exactly the recommended follow‐up, by health care provider who took the screening test (GP vs. gynecologist) and the woman's history of screening abnormalities in the past 15 years (none vs. at least one abnormal test). These models were mutually adjusted. The numbers of tests and diagnostic procedures per throughout the entire follow‐up were reported with means and fifth to 95th percentile ranges. Statistical analyses were performed using STATA version 16.1.

### 2.7 Sensitivity analysis

In the main analysis, we evaluated the adherence to the first three follow‐up visits. This was to avoid bias due to misclassification of women whose follow‐up was censored at 4 years. We then performed a sensitivity analysis to investigate how the adherence to the recommendations was affected by including the remaining tests and procedures throughout the entire follow‐up pathway.

## 3 RESULTS

**FIGURE 3:**
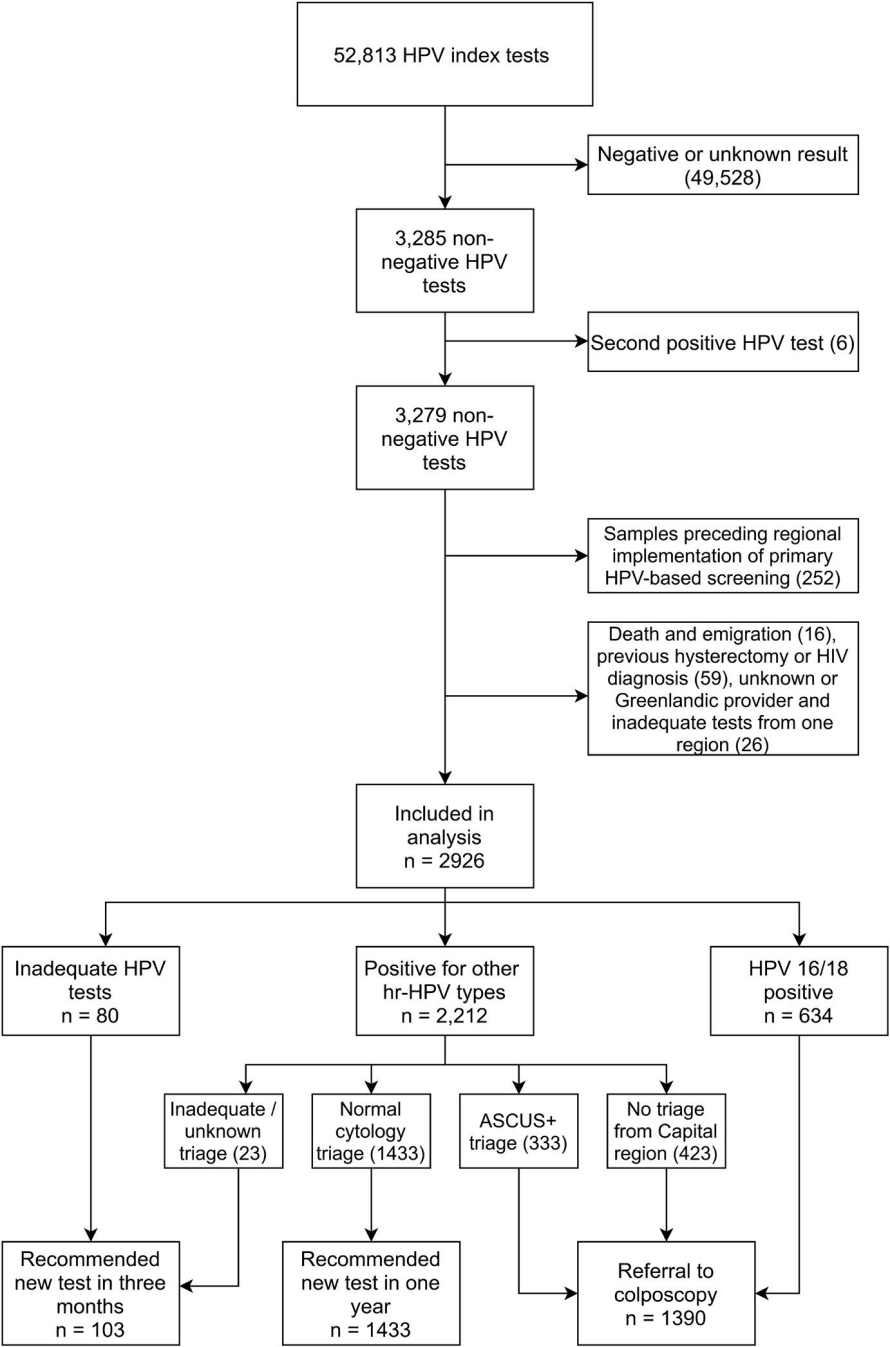
Inclusion and exclusions in the study

We identified 52,813 women with HPV tests that fulfilled our definition of a screening test. Of those, 3285 (6.2%) had a nonnegative screening test result. After exclusions described in Figure 3, 2926 women were included in the final analysis. Of these, 80 had inadequate HPV tests, 634 were positive for HPV16/18, and 2,212 were positive for other high‐risk HPV genotypes or for unknown genotypes if the woman were from the Capital region where HPV genotyping was not reported. The proportions of women with a recommendation for follow‐up in 1 year and a colposcopy referral were very similar (49% vs. 48%, respectively), while 4% were recommended for a new test in 3 months (Figure 3). As shown in Table 1, 10% of screening tests were taken by a gynecologist, either in private practice or in a hospital setting, while 16% of women had one or more abnormal screening tests in the preceding 15 years (Table 1).

**TABLE 1:** Adherence to the recommendations during the first three follow‐up visits after the index HPV test, by follow‐up recommendation following the index test, administrative region, history of screening abnormalities, and type of screening provider

	No follow‐up *N* (%) [95% CI]	Less than recommended^a^ *N* (%) [95% CI]	Exactly as recommended *N* (%) [95% CI]	More than recommended^b^ *N* (%) [95% CI]	Total *N* (%)
Recommended follow‐up after primary screening test					
New test in 3 months	7 (6.8) [2.8–13.5]	38 (36.9) [27.6–47.0]	30 (29.1) [20.6–38.9]	28 (27.2) [18.9–36.8]	103 (100)
Referral to colposcopy	24 (1.7) [1.1–2.6]	587 (42.2) [39.6–44.9]	367 (26.4) [24.1–28.8]	412 (29.6) [27.2–32.1]	1390 (100)
New HPV test in 1 year	88 (6.1) [5.0–7.5]	744 (51.9) [49.3–54.5]	350 (24.4) [22.2–26.7]	251 (17.5) [15.6–19.6]	1433 (100)
Administrative region					
Capital	8 (1.9) [0.8–3.4]	209 (49.4) [44.5–54.3]	82 (19.4) [15.7–23.5]	124 (28.8) [25.0–33.9]	423 (100)
Central	20 (3.5) [2.1–5.3]	292 (50.5) [46.4–54.7]	135 (23.4) [20.0–27.0]	131 (22.7) [19.3–26.3]	578 (100)
Southern	33 (6.1) [4.2–8.4]	254 (46.9) [42.6–51.2]	138 (25.5) [21.8–29.3]	117 (21.6) [18.2–25.3]	542 (100)
Zealand	34 (3.7) [2.6–5.2]	419 (45.9) [42.6–49.2]	277 (30.3) [27.4–33.4]	183 (20.0) [17.5–22.8]	913 (100)
Northern	24 (5.1) [3.3–7.5]	195 (41.5) [37.0–46.1]	115 (24.5) [20.6–28.6]	136 (28.9) [24.9–33.3]	470 (100)
Abnormal screening tests in last 15 years^c^					
None	103 (4.2) [3.4–5.1]	1131 (46.1) [44.1–48.1]	644 (26.3) [24.5–28.0]	575 (23.4) [21.8–25.2]	2453 (100)
One or more	16 (3.4) [1.9–5.4]	238 (50.3) [45.7–54.9]	103 (21.8) [18.1–25.8]	116 (24.5) [20.7–28.7]	473 (100)
Screening provider					
General practitioner	105 (4.0) [3.3–4.8]	1234 (46.9) [45.0–48.8]	679 (25.8) [24.1–27.5]	613 (23.3) [21.7–25.0]	2631 (100)
Gynecologist	14 (4.8) [2.6–7.8]	135 (45.8) [40.0–51.6]	68 (23.1) [18.4–28.3]	78 (26.4) [21.5–31.9]	295 (100)
Total	119 (4.1) [3.4–4.8]	1369 (46.8) [45.0–48.6]	747 (25.5) [24.0–27.2]	691 (23.6) [22.1–25.2]	2926 (100)

Abbreviation: HPV, human papillomavirus.

^a^ The follow‐up was (partially) insufficient.

^b^ Women received more follow‐up tests or treatments than recommended.

^c^ The 15‐year period includes the 2 years prior to the screening test during which no abnormal tests were registered.

### 3.1 Adherence to the first three recommended follow‐up visits

The adherence to the recommended care during the first three follow‐up visits was 26% (95% CI: 24%–27%) in total (Table 1). Four percent of women had no follow‐up at all registered for a period of up to 2 years after screening, and 47% (95% CI: 45%–49%) received less than what the national guidelines recommended. Among the 691 women with more extensive follow‐up than recommended, 45 women, ~7% (95% CI: 5%–9%; i.e., just under 2% of all studied women) ultimately had a CIN2+ diagnosis (data not shown).

### 3.2 Factors associated with deviations from the recommended first three follow‐up visits

Women recommended for a new test in 3 months had a higher risk of insufficient follow‐up if their screening test was taken by a gynecologist, with RR_adj_ of 1.41 (95% CI: 1.09–1.83; Table 2). In contrast, the risk of excessive follow‐up was increased with a gynecologist as a screening provider in women with a recommendation for a new test in 1 year, RR_adj_:1.32 (95% CI: 1.04–1.68). The risk of having a follow‐up less than recommended was also significantly higher in women with a history of one or more screening abnormalities if they were recommended for a new test in 3 months or 1 year, RR_adj_: 1.72 (1.23–2.41) and RR_adj_: 1.11 (95% CI: 1.01–1.23), respectively (Table 2).

**TABLE 2:** Adjusted relative risks (RR's) of deviation from the recommended follow‐up, compared to having exactly the recommended follow‐up, by screening provider, the woman's 15‐year history of abnormal tests, and the type of the recommended follow‐up

	No follow‐up within 2 years RR (95% CI)	Less than recommended^a^ RR (95% CI)	Insufficient follow‐up combined^b^ RR (95% CI)	More than recommended^c^ RR (95% CI)
*New test in 3 months*				
Previous screening abnormalities^d^				
None	1	1	1	1
One or more	n/a	**1.72 (1.23–2.41)**	**1.53 (1.15–2.03)**	1.60 (0.92–2.78)
Screening provider				
General practitioner	1	1	1	1
Gynecologist	3.10 (0.87–11.0)	**1.41 (1.07–1.85)**	**1.41 (1.09–1.83)**	1.12 (0.50–2.50)
*Referral to colposcopy*				
Previous screening abnormalities^d^				
None	1	1	1	1
One or more	0.52 (0.13–2.16)	1.04 (0.92–1.19)	1.03 (0.91–1.17)	1.06 (0.89–1.27)
Screening provider				
General practitioner	1	1	1	1
Gynecologist	2.05 (0.74–5.63)	1.14 (1.00–1.31)	1.15 (1.00–1.31)	1.00 (0.79–1.26)
*New HPV test in 1 year*				
Previous screening abnormalities^d^				
None	1	1	1	1
One or more	1.19 (0.71–1.97)	**1.11 (1.01–1.23)**	1.10 (1.00–1.20)	1.11 (0.87–1.41)
Screening provider				
General practitioner	1	1	1	1
Gynecologist	0.91 (0.47–1.76)	0.87 (0.74–1.04)	0.89 (0.76–1.04)	**1.32 (1.04–1.68)**

Statistical significant estimates presented in bold.

Abbreviation: HPV, human papillomavirus.

^a^ The follow‐up was insufficient based on the first three visits after screening.

^b^ The categories of “No follow‐up” and “Less than recommended” combined.

^c^ The women received more follow‐up tests or treatment than recommended.

^d^ In the last 15 years, including the 2 years prior to the screening test, during which women were required to have had no abnormalities.

### 3.3 Providers and procedures throughout the entire follow‐up pathway

Among women recommended a referral to colposcopy directly after the screening test, 96% were seen by a gynecologist as recommended (Table 3). Among these women who attended a gynecological appointment, 88% had at least one biopsy as recommended, or a conization (Table 4). Throughout the rest of the follow‐up, the proportions with gynecologist visits varied between 70% and 82% in this group. Among women who were still undergoing follow‐up at the specific time points, the proportions undergoing treatment did not decrease over time (Table 4).

**TABLE 3:** Provider distribution throughout the follow‐up, by follow‐up recommendation following the primary screening test. The table includes women with at least one follow‐up test

	Recommended follow‐up after primary screening test		
	New test in 3 months, *N* (%)	Referral to colposcopy, *N* (%)^a^	New HPV test in 1 year, *N* (%)^b^
Proportion with timely first follow‐up	39 (40.6)	1231 (90.1)	765 (56.9)
First follow‐up			
General practitioner	58 (60.4)	50 (3.7)	>1134 (84.5)
Gynecologist	38 (39.6)	1316 (96.3)	>205 (15.5)
Second follow‐up			
General practitioner	(34.5)	222 (20.9)	372 (44.9)
Gynecologist	(65.5)	838 (79.1)	457 (55.1)
Third follow‐up			
General practitioner	(41.2)	165 (26.7)	284 (47.3)
Gynecologist	(58.8)	453 (73.3)	316 (52.7)
Fourth follow‐up			
General practitioner	(42.9)	100 (24.9)	203 (50.5)
Gynecologist	(57.1)	301 (75.1)	199 (49.5)
Fifth follow‐up			
General practitioner	n/a^c^	74 (26.7)	99 (42.3)
Gynecologist	n/a^c^	203 (73.3)	135 (57.7)
Sixth follow‐up			
General practitioner	n/a^c^	52 (29.4)	47 (35.9)
Gynecologist	n/a^c^	125 (70.6)	84 (64.1)
Seventh follow‐up			
General practitioner	n/a^c^	21 (18.9)	22 (30.1)
Gynecologist	n/a^c^	90 (81.1)	51 (69.9)
Eighth follow‐up			
General practitioner	n/a^c^	10 (17.9)	10 (27.0)
Gynecologist	n/a^c^	46 (82.1)	27 (73.0)

Note

The table only shows the first 8 follow‐up visits, although some women had up to 11 follow‐up visits.

Abbreviation: HPV, human papillomavirus.

^a^ Screening tests positive for HPV 16/18 or for other high‐risk HPV genotypes combined with cytological abnormalities on triage.

^b^ Screening tests positive for other high‐risk HPV genotypes combined with normal cytology on triage.

^c^ Due to data restrictions (potential for re‐identification) we could not report results for categories including fewer than five women.

**TABLE 4:** Procedures at each follow‐up visit, by follow‐up recommendation after the primary screening test. The table includes women with at least one follow‐up test. Follow‐up procedures were classified according to the most invasive registered procedure

	Recommended follow‐up after the primary screening test		
	New test in 3 months, *N* (%)	Referral to colposcopy, *N* (%)^a^	New HPV test in 1 year, *N* (%)^b^
First follow‐up			
Only cytology	79 (82.3)	170 (12.5)	>1263 (~94.0)
Biopsy	17 (17.7)	1150 (84.2)	>73 (~5.0)
Conization	–	46 (3.4)	n/a^c^
Second follow‐up			
Only cytology	(69.0)	660 (62.3)	474 (57.2)
Biopsy	(17.2)	242 (22.8)	331 (39.9)
Conization	(13.8)	158 (14.9)	24 (2.9)
Third follow‐up			
Only cytology	(82.4)	402 (65.1)	421 (70.2)
Biopsy	(17.7)	163 (26.4)	137 (22.8)
Conization	n/a^c^	53 (8.6)	42 (7.0)
Forth follow‐up			
Only cytology	n/a^c^	248 (61.9)	312 (77.6)
Biopsy	n/a^c^	107 (26.7)	59 (14.7)
Conization	n/a^c^	46 (11.5)	31 (7.7)
Fifth follow‐up			
Only cytology	n/a^c^	186 (67.2)	170 (72.7)
Biopsy	n/a^c^	61 (22.0)	41 (17.5)
Conization	n/a^c^	30 (10.8)	23 (9.8)
Sixth follow‐up			
Only cytology	n/a^c^	117 (66.1)	81 (61.8)
Biopsy	n/a^c^	39 (22.0)	33 (25.2)
Conization	n/a^c^	21 (11.9)	17 (13.0)
Seventh follow‐up			
Only cytology	n/a^c^	74 (66.7)	45 (61.6)
Biopsy	n/a^c^	16 (14.4)	20 (27.4)
Conization	n/a^c^	21 (18.9)	8 (11.0)
Eighth follow‐up			
Only cytology	n/a^c^	30 (53.6)	21 (56.8)
Biopsy	n/a^c^	19 (33.9)	9 (24.3)
Conization	n/a^c^	7 (12.5)	7 (18.9)

Note

The table only shows the first 8 follow‐up visits, although some women had up to 11 follow‐up visits.

Abbreviation: HPV–human papillomavirus.

^a^ Screening tests positive for HPV 16/18 or for other high‐risk HPV genotypes combined with cytological abnormalities on triage.

^b^ Screening tests positive for other high‐risk HPV genotypes combined with normal cytology on triage.

^c^ Due to data restrictions (potential for re‐identification) we could not report results for categories including fewer than five women.

Overall, 72% had at least one gynecologist visit during their entire follow‐up and of those 26% (19% of all HPV‐positive women, and just over 1% of all screened women) underwent conization at some point (data not shown).

### 3.4 Sensitivity analysis of adherence to recommended follow‐up throughout the entire follow‐up

When we included all available data in the analysis, the proportion of women who received exactly the recommended follow‐up was slightly lower than in the main analysis, 22%, and the proportion of women having insufficient care was slightly higher, 50% (Appendix B, Table B1). The median time spent in follow‐up was 489 days (IQR: 237–1006), counted from the screening test onward. Among women screened in 2012 or 2013 (*n* = 398), who had more than 7 years of follow‐up in our data, the median time spent in follow‐up was 528 days (IQR: 350–1129). The follow‐up lasted more than 4 years in approximately 17% of the women, and 7 or more years in 1% (data not shown). Other results showed similar patterns as in the main analyses (Appendix B, Tables B2 and B3).

### 3.5 Women with three positive HPV tests and normal cytological/histological findings

In our study population, 235 (8%) women had three or more consecutive positive HPV tests combined with normal cytology or histology (data not shown). Their median follow‐up time was 1631 days (IQR: 1459–1981; data not shown). This is, however, underestimated as some women had only up to 4 years of follow‐up in our data; for women screened in 2012 or 2013 (*n* = 37), the median was 2225 days (IQR: 1730–2394). These persistently HPV‐positive women were less likely to have their follow‐up in accordance with the recommendations. Fifteen percent (95% CI: 10%–20%) had exactly the recommended follow‐up during the first three follow‐up visits (data not shown), compared to 26% in the entire study population (see above).

### 3.6 Number of follow‐up tests and procedures throughout the entire follow‐up

Interestingly, having insufficient follow‐up did not contribute to fewer diagnostic procedures (Table 5). Among women recommended for a new test in 3 or 12 months, more HPV, cytology, and histological procedures were performed if they had insufficient follow‐up compared to women who had follow‐up in accordance with the recommendations. For every 100 women 29 had a conization if they were referred for a colposcopy directly after a screening test; among those who were recommended a new test in 3 months, six out of 100 women had a conization, and 12 out of 100 women after being recommended a new test in 12 months (Table 5).

**TABLE 5:** Follow‐up procedures performed during the entire follow‐up pathway, by adherence during the first three visits and the follow‐up recommendation after the index test

	Less than recommended Mean (5th–95th percentile)	Exactly as recommended Mean (5th–95th percentile)	More than recommended Mean (5th–95th percentile)	Total Mean (5th–95th percentile)
New test in 3 months				
HPV tests	1.74 (0–8)	1.20 (1–2)	1.14 (0–3)	1.40 (0–5)
Cytological tests	2.17 (0–8)	1.20 (1–2)	1.43 (0–4)	1.58 (0–5)
Histological tests	0.74 (0–3)	–	0.14 (0–1)	0.33 (0–2)
Conizations	0.16 (0–1)	–	–	0.06 (0–1)
Referral for colposcopy				
HPV tests	1.07 (0–4)	1.35 (0–5)	1.31 (0–5)	1.22 (0–5)
Cytological tests	1.81 (0–6)	2.24 (0–7)	2.37 (0–7)	2.09 (0–6)
Histological tests	1.12 (0–3)	1.45 (1–3)	1.53 (0–3)	1.33 (0–3)
Conizations	0.13 (0–1)	0.51 (0–1)	0.32 (0–1)	0.29 (0–1)
New HPV test in 1 year				
HPV tests	2.33 (1–5)	1.52 (1–4)	2.04 (0–6)	2.06 (1–5)
Cytological tests	2.69 (1–6)	1.81 (1–5)	2.59 (0–7)	2.44 (0–6)
Histological tests	0.48 (0–2)	0.37 (0–2)	0.88 (0–2)	0.53 (0–2)
Conizations	0.12 (0–1)	0.11 (0–1)	0.12 (0–1)	0.12 (0–1)

Abbreviation: HPV, human papillomavirus.

## 4 DISCUSSION

### 4.1 Main findings

Among Danish women with a nonnegative HPV test result in their last program screening round, only a quarter received the follow‐up as recommended by the national guidelines. Almost half of the women received insufficient follow‐up, either because test types were incorrect, or the tests were used with longer than recommended delays. Insufficient follow‐up was not associated with a less frequent use of HPV or cytology tests and biopsies, since the numbers of procedures were on average higher than among women who followed the recommended follow‐up pathways. Furthermore, follow‐up pathways tended to span fairly long periods, with about 17% of HPV‐positive women continuing to receive additional tests for longer than 4 years. Women with persistently positive HPV tests but without detectable lesions, which is the group where the uncertainty regarding the clinical recommendations remains high, had particularly long pathways.

### 4.2 Strengths and limitations

Completeness of data from the Danish health care registries is considered high.^22^ These registers allowed us to include an unselected study population, within which we could reconstruct the entire clinical management pathways because data could be linked at the individual level. Even though we included all women satisfying the inclusion criteria, some of the subgroups were nevertheless small. This precluded more detailed analyses, for example, on the association between guideline adherence and screening provider and administrative region. Another limitation of the study may be that we categorized the women into a specific adherence category immediately after a deviation occurred; for example, women who were delayed with their follow‐up were categorized as having “less than recommended.” Some of them might have indeed ended up having more excessive care later in their pathway. The opposite may have also taken place. This categorization was used to keep the study manageable.

### 4.3 Comparison with other studies and clinical implications

We previously reported findings from a similar study including Danish women who continued to be screened with cytology as the primary screening method at the age of 23–59. That cohort and the one described here were screened during the same calendar period. Nevertheless, the overall proportion of women following the recommended follow‐up was substantially lower in the current study, 26% versus 43% among younger women.^12^ It is unclear what caused this difference, but age can probably not explain it. In cytology screening, older women seem to have the highest levels of adherence with the recommended follow‐up,^3^, ^5^, ^23^, ^24^, ^25^ although the results vary by screening diagnosis.^5^, ^6^, ^26^ This was also true in our cytology screening study, where the oldest included women, that is, those aged 50–59 years, generally had better adherence than younger women, particularly if their screening diagnosis was not severely abnormal.^12^

Using these registry data, we could not determine whether the deviations from the recommendations were caused by women's preferences or by deviations at provider or organizational level. For women, both undergoing either a colposcopy or repeated testing is distressing.^27^, ^28^, ^29^ Previous cytology‐based studies have found that when given the choice between a direct colposcopy and surveillance, a majority of women prefer the former, even in case of mild abnormalities.^30^ This was confirmed in a study of older Danish women undergoing HPV testing, where women seemed tolerant toward direct treatment instead of surveillance, even if that means over‐treatment.^31^ It is possible that screening providers have not, particularly in the beginning period, correctly implemented the new guidelines, potentially with insufficient capacity at gynecological services as an explanation. We performed a sensitivity analysis (not tabulated), excluding the first 6 months of the implementation in each region, but the overall patterns of adherence to the guidelines and health care resource use did not change. A more likely explanation could be that the national clinical management guidelines, which are to some extent open‐ended, may be interpreted differently among the individual gynecologists and may motivate to a more individualized approach to the woman's follow‐up, particularly in women with persistently positive HPV tests without a treatable lesion. In women with nonrepresentative biopsies, which is a common problem at this age, open‐ended guidelines may have resulted in post‐colposcopic surveillance with cytological testing instead of with biopsies.

Receiving less than the recommended follow‐up did not reduce the overall number of follow‐up tests and other procedures in this screening program. On average, this group of women in fact underwent more cytology and HPV testing than did women with exactly the recommended follow‐up. This could be potentially explained with compensatory procedures to make up for the delays in providing care, as we observed in cytology‐based screening.^12^ In the Capital region, on the other hand, more biopsies were taken on average (1.33 vs. 0.84) and fewer cytology (1.26 vs. 2.41) and HPV tests (0.52 vs. 1.82) than in other regions (Appendix B, Table B4). This is not surprising, as the region used a colposcopy referral for all women with a positive HPV test. Colposcopies with biopsies occupy many more resources such as time, personnel, and equipment than do HPV and cytology tests. Consequently, the overall resources used to complete the follow‐up after a positive exit HPV test varied to a great extent between the regions. This is probably not unusual, and we consider it to be plausible that other countries would also see regional variation in resource use due to local practices.

A very high proportion of women with a recommendation for colposcopy was registered with a first visit taking place at a gynecologist, our proxy for a colposcopy (96%, Table 3). A high adherence to follow‐up after the most severe screening abnormalities is in line with findings from other studies.^7^, ^8^, ^9^, ^32^ However, only 91% of these women had a biopsy or conization, which means that 9% had no histological sampling at the gynecological visit. The corresponding proportion among younger women, derived from our cytology study on women aged 23–59, was 6%.^12^ Up to 65% of colposcopies among women older than 60 years are inadequate due to a non‐visible or only partly visible transformation zone.^33^, ^34^, ^35^ The challenge of taking representative cervical biopsies among older women and the fact that these women are not scheduled to receive a new screening invitation may help explain why women end up having more tests than recommended. Hence, their follow‐up pathways are sometimes very long. We estimated that 17% of HPV‐positive women in this age group were in follow‐up for longer than 4 years. The even longer follow‐up pathways among women with persistently positive HPV tests with a negative cytology test and/or biopsy underscore the need for more explicit evidence‐based guidelines for these women. Although the prevalence of CIN2+ might be very low in these women,^34^ clinicians might still be reluctant to discharge them from the screening program for fear of a residual risk of cervical cancer. Recently published results from a study in which Danish women older than 70 were invited for a single HPV screen showed that 4.1% of the women were HPV‐positive and 7.5% of those had CIN3+.^17^

The revised guidelines for the management of HPV‐positive women aged 60 and over published in 2019^36^ recommend "considering an immediate conisation" in case of poor visibility of the transformation zone. This might result in fewer long surveillance pathways in the future. However, the challenge with women having persistent HPV infections and no cytological/histological abnormalities in adequate cervical samples remain, and the guidelines remain open‐ended in that aspect. The follow‐up pathways of these women need to be systematically monitored, in order to lower the risk of creating highly individualized pathways with poor quality and higher resource use.

## 5 CONCLUSION

Overall, the adherence to the Danish national recommendations for follow‐up after a positive HPV test in the last screening round at 60–64 years has been poor. Moreover, these women have very long follow‐up pathways and a non‐negligible proportion undergo tests and procedures more than 4 years after screening. This indicates an urgent need for guidelines with more explicit recommendations for follow‐up tests, treatments and termination of screening, and underscores the importance of systematically monitoring of the follow‐up pathways after screening. Further research on how disease outcomes are associated with adherence to follow‐up after screening is needed.

## CONFLICT OF INTEREST

Susanne Fogh Jørgensen reports no conflict of interest. Berit Andersen received HPV tests from Roche and self‐sampling kits for HPV self‐sampling for other studies. The department received a grant from Helsefonden during this study. Lone Kjeld Petersen reported no conflict of interest. Matejka Rebolj received funding from Public Health England for the epidemiological evaluation of the English HPV pilot; is a member of the Public Health England Laboratory Technology Group and HPV Self‐sampling Operational Steering Group and Project Board; attended meetings with various HPV assay manufacturers; her employer received a fee for lecture from Hologic on her behalf. Sisse Helle Njor reports a grant from Helsefonden during this study.

## RESEARCH ETHICS

According to EU's General Data Protection Regulation (article 30), the project was listed at the record of processing activities for research projects in Central Denmark Region (J. no.: 1‐16‐02‐301‐18). According to Danish legislation, notification of register‐based research projects to the research ethics committee system is only required if the project involves human biological material. Therefore, this study may be conducted without an approval from the Ethics Committees.

## Supporting information

Supplementary Materials

Supplementary Materials

## Data Availability

The data that support the findings of this study are available from The Danish Health Data Authority. Restrictions apply to the availability of these data, which were used under license for this study. Data may be available upon reasonable request to The Danish Health Data Authority with permission.^37^
