# Supplementary material for: Adherence to follow‐up after the exit cervical cancer screening test at age 60–64: A nationwide register‐based study
Source: Cancer Med. 2021 Nov 12;11(1):224–37. doi: 10.1002/cam4.4420 (PMC8704149; doi:10.1002/cam4.4420)
Supplement: Supplementary file 1 — Supplementary Materials [file CAM4-11-224-s001.docx]

**Appendix A. Supplementary material for the method**


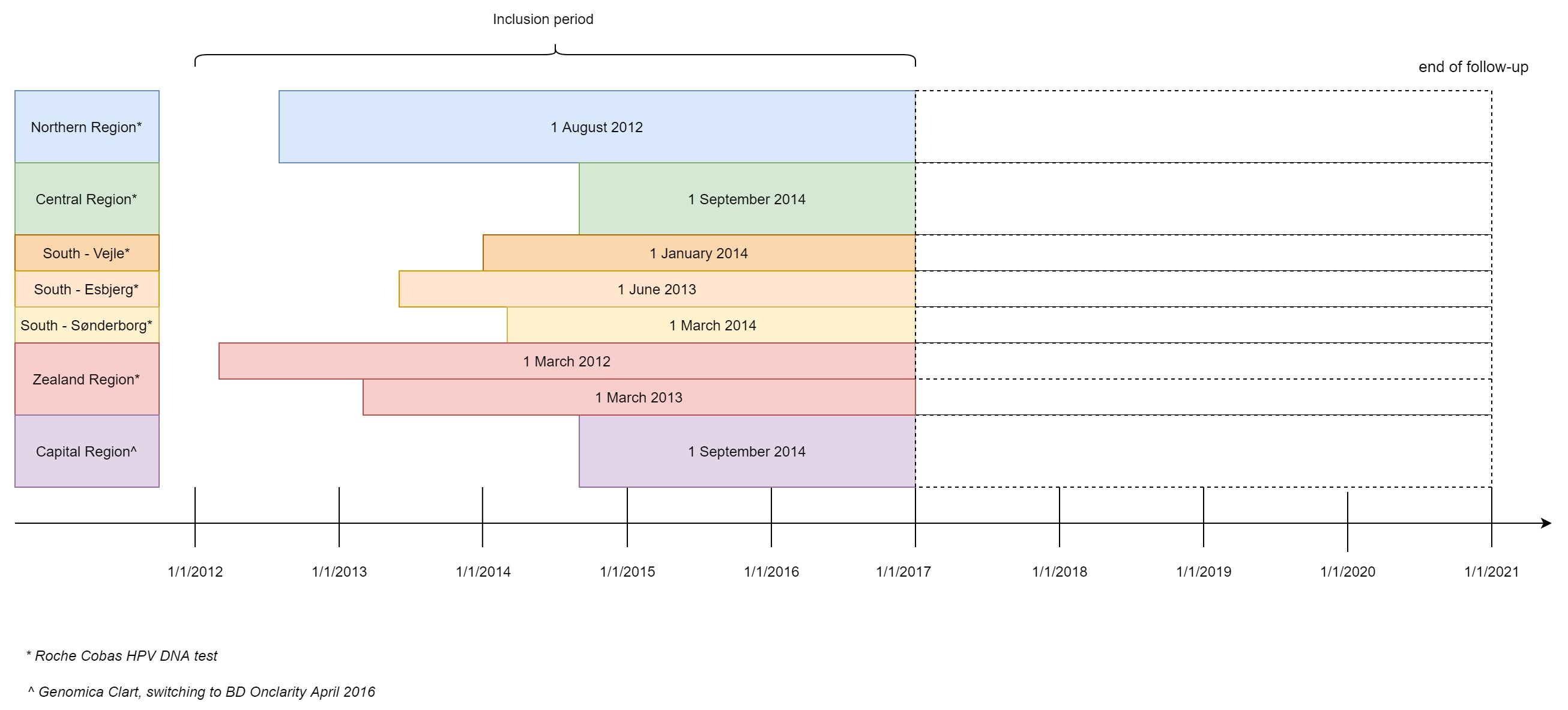


**Figure A1** Inclusion of women in the study, depending on when their administrative region began implementing HPV-based primary screening for women aged 60–64. (Information retrieved directly from contact persons at the administrative offices in each region.)


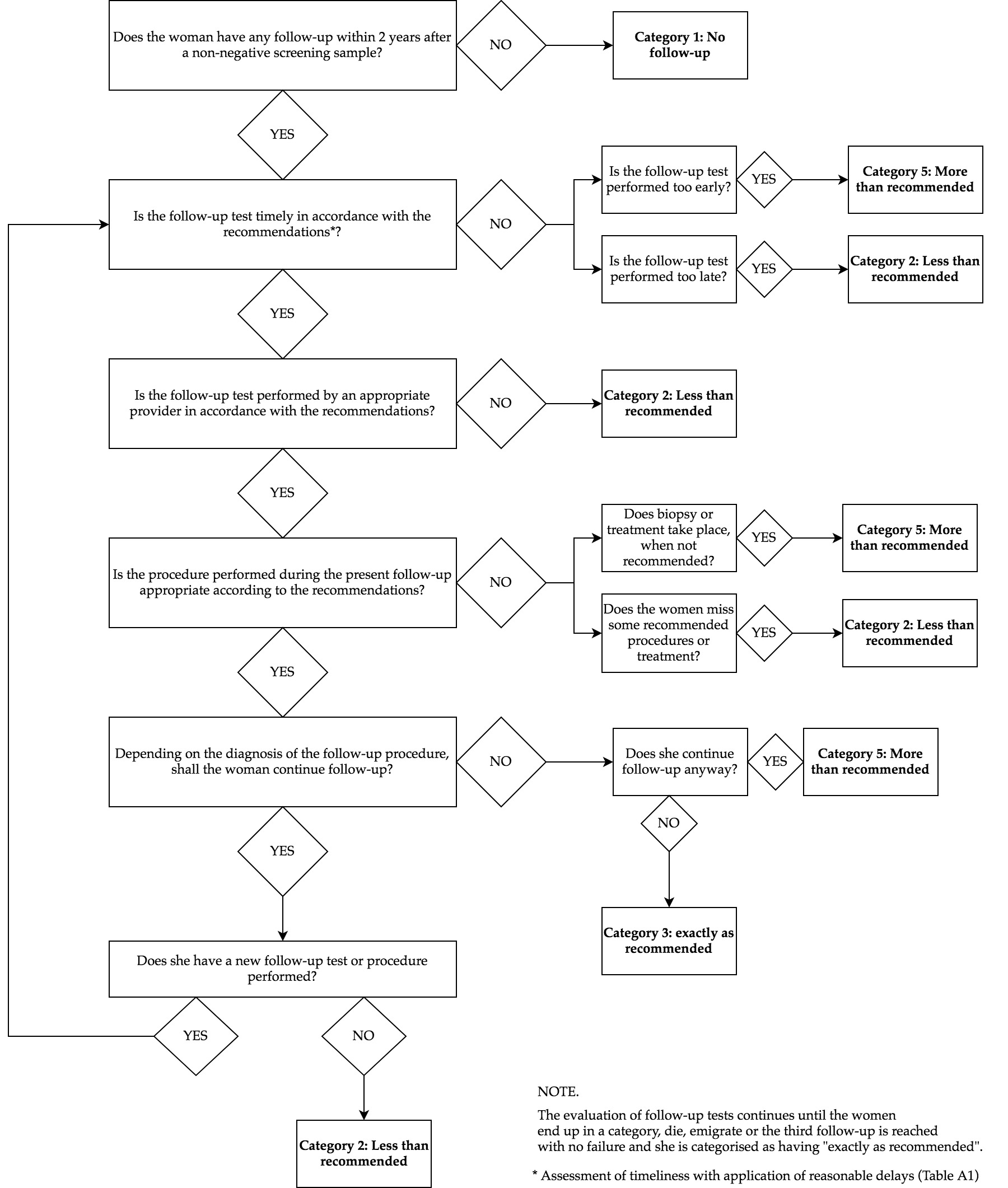


**Figure A2** Decision tree on categorization of women according to their adherence to the recommended follow-up

**Table A1** Timeliness of follow-up tests according to the national recommendations, including the reasonable delay accepted in the assessment of the adherence.

| **National recommended time limits of follow-up:** | **Accepted as adherence to recommendations*:** |
| --- | --- |
| Referral to colposcopy/gynecologist, that is, HPV 16/18 or ASCUS+ triage | Within 6 months /4 months** |
| New test in 3 months, that is, inadequate cytology or HPV tests | Within 2–6 months |
| New test in 6 months, that is, colposcopies with normal biopsies/CIN1 (if screening cytology was HSIL+) or inadequate biopsies. | Within 3–9 months |
| New test in 12 months, that is, normal triage or normal biopsies and CIN1 after first colposcopy (if screening cytology is not HSIL+) | Within 9–15 months |

* Follow-up tests performed earlier than the lower time boundary are categorized as too early (too much, with no health gain), follow-up tests performed later than the upper time boundary are categorized as too late (less than recommended)

** Four months only when evaluating the first follow-up

Abbreviations. HPV–human papilloma virus, ASCUS+ -–atypical squamous cells of undetermined significance or worse, the latter including low-grade squamous intraepithelial lesions (LSIL), atypical squamous cells, possibly high-grade (ASC-H), atypical glandular cells (AGC), or high-grade squamous intraepithelial lesions (HSIL), adenocarcinoma in situ and all carcinomas. CIN1–cervical intraepithelial neoplasia grade 1, HSIL+ - possibly high-grade (ASC-H), atypical glandular cells (AGC), or high-grade squamous intraepithelial lesions (HSIL), adenocarcinoma in situ and all carcinomas
