# Supplementary material for: Adherence to follow‐up after the exit cervical cancer screening test at age 60–64: A nationwide register‐based study
Source: Cancer Med. 2021 Nov 12;11(1):224–37. doi: 10.1002/cam4.4420 (PMC8704149; doi:10.1002/cam4.4420)
Supplement: Supplementary file 2 — Supplementary Materials [file CAM4-11-224-s002.docx]

Appendix B. Sensitivity analysis including an assessment of adherence throughout the entire follow-up pathways.

**Table B1**. Adherence to the recommendations during the entire follow-up pathway after the index HPV test, by follow-up recommendation following the index test, administrative region, 15-year history of screening abnormalities, and type of screening provider.

|  | **No follow-up in 2 years**  **N (%) [95%CI]** | **Less than recommended^a^**  **N (%) [95%CI]** | **Exactly as recommended**  **N (%) [95%CI]** | **More than recommended^b^**  **N (%) [95%CI]** | **Total** |
| --- | --- | --- | --- | --- | --- |
| **Screening diagnosis** |  |  |  |  |  |
| New test in 3 months | 7 (6.8) [2.8–13.5] | 39 (37.9) [28.5–48.0] | 28 (27.2) [18.9–36.8] | 29 (28.2) [19.7–37.9] | 103 (100) |
| Direct referral to colposcopy | 24 (1.7) [1.1–2.6] | 651 (47.0) [44.3–49.6] | 314 (22.7) [20.5–25.0] | 397 (28.6) [26.3–31.1] | 1386 (100) |
| New test in 1 year | 88 (6.1) [5.0–7.5] | 774 (54.0) [51.4–56.6] | 309 (21.6) [19.5–23.8] | 262 (18.3) [16.3–20.4] | 1433 (100) |
| **Administrative regions** |  |  |  |  |  |
| Capital | 8 (1.9) [0.8–3.7] | 229 (54.4) [49.5–59.2] | 81 (19.2) [15.6–23.3] | 103 (24.5) [20.4–28.9] | 421 (100) |
| Central | 20 (3.5) [2.1–5.3] | 303 (52.4) [48.3–56.6] | 124 (21.5) [18.2–25.0] | 131 (22.7) [19.3–26.3] | 578 (100) |
| Southern | 33 (6.1) [4.2–8.4] | 276 (50.9) [46.6–55.2] | 113 (20.9) [17.5–24.5] | 120 (22.1) [18.7–25.9] | 542 (100) |
| Zealand | 34 (3.7) [2.6–5.2] | 454 (49.8) [46.5–53.1] | 227 (24.9) [22.1–27.9] | 196 (21.5) [18.9–24.3] | 911 (100) |
| Northern | 24 (5.1) [3.3–7.5] | 202 (43.0) [38.5–47.6] | 106 (22.6) [18.9–26.6] | 138 (29.4) [25.3–33.7] | 470 (100) |
| **Previous screening abnormalities^c^** |  |  |  |  |  |
| None | 103 (4.2) [3.4–5.1] | 1217 (49.7) [47.7–51.7] | 557 (22.7) [21.1–24.5] | 572 (23.3) [21.7–25.1] | 2449 (100) |
| One or more | 16 (3.4) [2.0–5.5] | 247 (52.2) [47.8–57.0] | 92 (19.5) [16.0–23.4] | 116 (24.5) [20.8–28.8] | 471 (100) |
| **Provider of the screening test** |  |  |  |  |  |
| General practitioner | 105 (4.0) [3.3–4.8] | 1319 (50.2) [48.3–52.1] | 589 (22.4) [20.8–24.1] | 614 (23.4) [21.8–25.0] | 2627 (100) |
| Gynecologist | 14 (4.8) [2.6–7.8] | 145 (49.2) [43.3–55.0] | 62 (21.0) [16.5–26.1] | 74 (25.3) [20.2–30.4] | 295 (100) |
| **Total^d^** | **119 (4.1) [3.4–4.9]** | **1464 (50.1) [48.3–51.9]** | **651 (22.3) [20.8–23.8]** | **688 (23.5) [22.0–25.1]** | **2922 (100)** |

^a^ The follow-up was (partially) insufficient.

^b^ Women received more follow-up tests or treatments than recommended.

^c^ Period of 2 years prior to the screening (index) test, during which women were required to have had no abnormalities.

^d^ Four women are further excluded in the sensitivity analysis due to death or emigration during their follow-up pathway and could not be evaluated in terms of adherence to the entire follow-up.

**Table B2.** Adjusted relative risks (RR) of deviation from the recommended follow-up throughout the entire pathway, compared to having exactly the recommended follow-up, by screening provider, the woman’s 15-year history of abnormal tests, and the type of the recommended follow-up.

|  | **No follow-up within 2 years**  **RR (95%CI)** | **Less than recommended^a^**  **RR (95%CI)** | **Insufficient follow-up combined^b^**  **RR (95%CI)** | **More than recommended^c^**  **RR (95%CI)** |
| --- | --- | --- | --- | --- |
| **New test in 3 months** |  |  |  |  |
| **Previous screening abnormalities^d^** |  |  |  |  |
| None | 1 | 1 | 1 | 1 |
| One or more | n/a | **1.64 (1.19–2.27)** | **1.47 (1.07–1.77)** | 1.49 (0.86–2.59) |
| **Provider of the screening test** |  |  |  |  |
| General practitioner | 1 | 1 | 1 | 1 |
| Gynecologist | 2.90 (0.82–10.3) | **1.37 (1.05–1.79)** | **1.37 (1.12–1.94]** | 1.05 (0.44–2.46) |
| **Referral to colposcopy** |  |  |  |  |
| **Previous screening abnormalities^d^** |  |  |  |  |
| None | 1 | 1 | 1 | 1 |
| One or more | 0.49 (0.12–2.02) | 0.99 (0.88–1.12) | 0.99 (0.88–1.11) | 1.02 (0.86–1.22) |
| **Provider of the screening test** |  |  |  |  |
| General practitioner | 1 | 1 | 1 | 1 |
| Gynecologist | 1.90 (0.69–5.18) | 1.10 (0.97–1.24) | 1.09 (0.98–1.24) | 0.95 (0.74–1.21) |
| **New HPV test in 1 year** |  |  |  |  |
| **Previous screening abnormalities^d^** |  |  |  |  |
| None | 1 | 1 | 1 | 1 |
| One or more | 1.18 (0.71–1.94) | 1.09 (1.00–1.20) | 1.08 (0.99–1.18) | 1.10 (0.87–1.39) |
| **Provider of the screening test** |  |  |  |  |
| General practitioner | 1 | 1 | 1 | 1 |
| Gynecologist | 0.88 (0.46–1.70) | 0.88 (0.75–1.04) | 0.90 (0.78–1.04) | 1.22 (0.96–1.54) |

Abbreviations. HPV–human papillomavirus.

^a^ The follow-up was insufficient based on the first three visits after screening.

^b^ The categories of “No follow-up” and “Less than recommended” combined.

^c^ The women received more follow-up tests or treatment than recommended

^d^ In the last 15 years, including the 2 years prior to the screening test, during which women were required to have had no abnormalities.

**Table B3**. Follow-up procedures performed during the entire follow-up pathway, by adherence category and the follow-up recommendation after the index test.

|  | **Less than recommended**  **Mean**  **(5^th^–95^th^ percentile)** | **Exactly as recommended**  **Mean**  **(5^th^–95^th^ percentile)** | **More than recommended**  **Mean**  **(5^th^–95^th^ percentile)** | **Total Mean**  **(5^th^–95^th^ percentile)** |
| --- | --- | --- | --- | --- |
| **New in 3 months** |  |  |  |  |
| HPV tests | 1.82 (0–8) | 1.04 (1–2) | 1.17 (0–3) | 1.40 (0–5) |
| Cytological tests | 2.08 (0–8) | 1.04 (1–2) | 1.45 (0–4) | 1.58 (0–5) |
| Histological tests | 0.72 (0–3) | 0.00 | 0.14 (0–1) | 0.33 (0–2) |
| Conizations | 0.16 (0–1) | - | - | 0.06 (0–1) |
| **Referral to colposcopy** |  |  |  |  |
| HPV tests | 1.27 (0–5) | 0.93 (0–4) | 1.34 (0–5) | 1.22 (0–5) |
| Cytological tests | 2.06 (0–6) | 1.67 (0–5) | 2.47 (0–7) | 2.09 (0–6) |
| Histological tests | 1.20 (0–3) | 1.34 (1–3) | 1.54 (0–3) | 1.33 (0–3) |
| Conizations | 0.13 (0–1) | 0.59 (0–1) | 0.32 (0–1) | 0.29 (0–1) |
| **New HPV test in 1 year** |  |  |  |  |
| HPV tests | 2.38 (1–5) | 1.26 (1–3) | 2.08 (0–6) | 2.06 (1–5) |
| Cytological tests | 2.77 (1–6) | 1.41 (1–4) | 2.67 (0–7) | 2.44 (0–6) |
| Histological tests | 0.52 (0–2) | 0.23 (0–1) | 0.90 (0–2) | 0.53 (0–2) |
| Conizations | 0.12 (0–1) | 0.11 (0–1) | 0.11 (0–1) | 0.12 (0–1) |

Abbreviations. HPV–human papillomavirus.

**Table B4** Average numbers, including 5^th^–95^th^ percentile ranges, of follow-up procedures performed during the entire follow-up pathway in the Capital region and the other four regions combined.

|  | Capital region^a^,  Mean (5^th^–95^th^ percentile) | Other regions^b^,  Mean (5^th^–95^th^ percentile) |
| --- | --- | --- |
| HPV tests | 0.52 (0–3) | 1.82 (0–5) |
| Cytological tests | 1.26 (0–4) | 2.41 (0–6) |
| Histological tests | 1.33 (0–3) | 0.84 (0–3) |
| Conizations | 0.21 (0–1) | 0.20 (0–1) |

^a^ Capital Region referred all positive screening samples for colposcopy independent of the HPV genotype.

^b^ Northern region, Central region, Southern region, and Zealand region all followed the national recommendations of referring only HPV16/18 for colposcopy.
